# Pandemic Fatigue in Nursing Undergraduates: Role of Individual Resilience and Coping Styles in Health Promotion

**DOI:** 10.3389/fpsyg.2022.940544

**Published:** 2022-08-04

**Authors:** Rajesh Kumar, Kalpana Beniwal, Yogesh Bahurupi

**Affiliations:** All India Institute of Medical Sciences, Rishikesh, Rishikesh, India

**Keywords:** COVID-19, nursing, students, coping, resilience, fatigue

## Abstract

**Introduction:**

The COVID-19 pandemic was soon declared a global health threat and had significant economic and health implications. Unprecedented government measures brought massive shifts in teaching-learning pedagogy in nursing to curb the infection. The study was conducted to explore the predictors of pandemic fatigue among nursing undergraduates and mediating role of individual resilience and coping styles during the third wave in India.

**Methods:**

This online survey included 256 undergraduate nursing students studying at Tertiary Care Teaching Hospital in North India. Lockdown/Pandemic Fatigue Questionnaire, Brief Resilience Scale, and Coping Behavior Questionnaire were used to collect the information. Appropriate descriptive and inferential statistics were applied to compute the results.

**Results:**

Nursing undergraduates reported a moderate level of fatigue during the restrictions imposed at the time of the third wave. Students’ year of study (*p* = 0.001), tested positive during pandemic (*p* = 0.003), and post-COVID-19 hospitalization (*p* = 0.026) were found associated with higher fatigue status. Advanced age (*p* = 0.046) and higher personal resilience status (*p* < 0.001) were associated with lower fatigue levels. Resilience status (*ß* = − 4.311 *p* < 0.001) and second year of study (*ß* = 3.198, *p* = 0.015) were reported as independent predictors of pandemic fatigue in students.

**Conclusion:**

Findings suggest that lockdown-related fatigue was common in nursing undergraduates. Considering negative consequences on mental health, routine psychosocial screening of the nursing students should be conducted. Recommending stress-relieving measures should be enforced to help nursing undergraduates to combat lockdown-induced exhaustion.

## Introduction

The COVID-19 pandemic cases were detected in Wuhan in China’s Hubei province for 2 years (Aslan and Pekince, 2021). The COVID-19 pandemic was declared a global threat and significantly impacted each area of life, including health and economics (Labrague and Ballad, 2021). Every government has taken unprecedented measures to control further transmission, including mandatory lockdown, strict social distancing, frequent handwashing, and mandatory home quarantine or isolation after traveling to other parts or another country (Dharra and Kumar, 2021).

In India, the government imposed a countrywide lockdown and a partial lockdown named “night curfew” since the pandemic, forcing people to stay at home, reducing social interaction and physical activities, and leaving them with many psychosocial issues (Kumar et al., 2021). In addition, medical schools around the globe were also shut down before the pandemic reached on peak and left the students amid course completion dilemmas (Harries et al., 2021). However, government mitigating measures, including lockdown and social distancing, helped slow down the transmission of the virus but adversely affected the physical and psychological health and wellbeing of all categories of people, including nursing students (Labrague and Ballad, 2021).

Medical and nursing staff always take the forefront of any particular pandemic or outbreak and risk their lives. They are more prone and vulnerable to getting infection considering close contact with the infection, high stress, lack of rest, and extended duty hours in the hospital (Luberto et al., 2020). Extending course deadlines and duration might further intensify the stress and anxiety among nursing students (Huang et al., 2020). The changes in teaching-learning pedagogy and curriculum delivery of the nursing students might be expected to intensify students’ stress further. In addition, changes in the routine of academic, social restriction, and lifestyle behavior might negatively impact psychological wellbeing (Aslan and Pekince, 2021). Sadly, many health professionals lost their lives while providing care to patients with coronavirus at the hospital in the ongoing pandemic—every new strain of virus hammering the mental health of everyone and creating more panic among health professionals (Aslan and Pekince, 2021).

Resilience is a personality trait that helps an individual become strong and bounce back during a stressful situation or help to cope with adverse conditions (Hamadeh Kerbage et al., 2021). A higher resilience will help adapt to a stressful situation and help deal with mental, emotional, and educational challenges. Earlier literature reported the importance of resilience, ineffective adjustment, improving social relationships, social support, and psychological wellbeing (Yu et al., 2019). Indeed, resilience is considered one of the critical personality attributes affecting the retention of nursing students in their studies (Moloney et al., 2018).

Evidence shows that health professionals and university college students reported a higher incidence of psychological stress than the general population (Luberto et al., 2020). These students are more vulnerable to developing emotional problems, anxiety, stress, and depression considering fear of failure, time crisis, session duration change, exam pattern and timing, neglected self-care, and social restrictions to meeting family and friends (Dyrbye and Shanafelt, 2016; Yu et al., 2019). Huge uncertainties and confusion about the fixed deadline for the pandemic left the students with many questions related to their career selection and future (Agu et al., 2021). Still, there is no light on the other side of dark tunnels, and experts speculate vividly to end this pandemic.

Therefore, there is a pressing need to explore predictors of pandemic fatigue and understand the mediating role of resilience and coping on health after 2 years of the pandemic in nursing undergraduates.

## Materials and Methods

### Design, Sample, and Settings

This online cross-sectional survey was conducted after the end of the second wave of coronavirus in India when daily cases were reached below 10,000. At the same time, a new strain of COVID-19, Omicron, was knocking on the door to spread panic around the globe. The survey link was supplied with a consent form and asked each participant to provide consent before participating in the online survey. The survey questionnaire, Google Forms, was shared with 300 nursing students on the WhatsApp number of an individual nursing student, and 278 (92.66%) participants responded to the survey. Finally, after carefully scrutinizing the filled questionnaire, 256 questionnaires were deemed suitable for the analysis.

### Instrumentations

Three structured and standardized questionnaires were used in the study. The Pandemic/Lockdown Fatigue Scale, the Brief Resilience Scale, the Brief Coping Questionnaire, and structured socio-demographics datasheet are used to collect personal and professional information from the participants.

#### Socio-Demographic Sheet

It consists of information on age, year of study, experience as a bedside nurse, being infected with COVID-19 virus and quarantine status and days, admission to hospital after getting an infection, and loss of family members due to COVID-19 infection. A group of microbiology, nursing, and infectious disease experts was requested to validate the profile. The inclusion items were based on more than 80% expert consensus for the items’ relevancy.

#### Pandemic/Lockdown Fatigue Scale

This scale was used to assess the exhaustion level among nursing students associated with frequent preventive measures like quarantine, home isolation, and other government-imposed guidelines to stop the spread of the transmission of the COVID-19 virus. It is a ten-item Likert scale ranging from 1 (never) to 5 (always) with a maximum possible score of 50. The scale has an excellent criterion validity and acceptable concurrent validity in earlier similar work (Michielsen et al., 2003; Labrague and Ballad, 2021). The scale’s internal consistency was measured using Cronbach’s alpha and was 0.84 for the present study. The scale content validation sought by the experts in microbiology, nursing, and internal medicine and scale rated relevant and appropriate for the interest of the population. The test–retest validity of the scale was 0.82 in the present study.

#### Brief Resilience Scale

The scale was used to determine the participants’ ability to bounce back in unpleasant or difficult situations associated with pandemics and subsequent measures used to stop transmission during the pandemic. Students answered the scale by responding to a 5-point Likert scale ranging from 5 (describe me very well) to 0 (does not describe me at all). The scale is well validated in similar previous work (Smith et al., 2008; Labrague and Ballad, 2021), and in the present study, the internal consistency value of the scale was 0.88. The scale’s test–retest reliability was 0.89 in the present study.

#### Coping Behavior Questionnaire

The present study used a coping behavior questionnaire to measure the nursing undergraduates’ coping measures during a mandatory lockdown or restriction period. The students were requested to respond to this eight-item 5-point Likert scale ranging from 5 (strongly agree) to 1 (strongly disagree). The scale shows excellent criterion validity and acceptable reliability in earlier work reporting an internal consistency value of 0.85 (Savitsky et al., 2020; Labrague and Ballad, 2021). In the current study, the scale’s internal consistency was 0.83.

### Ethical Considerations

The study was approved by the Institutional Ethics Committee (AIIMS/IEC/21/642). The survey link also provided a consent form as a mandatory requirement to participate. However, the participants were ensured to protect privacy and confidentiality at each data collection stage and dissemination of the findings. Further, no personal information was obtained to protect the privacy and confidentiality of the students.

## Results

In this online survey, 256 nursing undergraduates studied in a nursing college associated with a teaching hospital were included. The mean age of the students was 21.27 years, with a standard deviation of 1.19 years. All the students were female, considering the admission to female students in the institute. A more significant number of students were from the second year (34.3%), followed by the third year (33.5%) and fourth year (32.3%). Since there was no admission for the new batch due to an ongoing pandemic, there was no participation from the first-year students. 39.3% of students were deployed as bed nurses during the pandemic and had experience as bedside nurses in the hospital. 11.3% of students were hospitalized after testing positive for COVID-19 (10.1%) during the ongoing pandemic.

Further, more than three fourth of students underwent quarantine (78.6%), with a mean duration of 13.27 days of quarantine. Sadly, 5.8% of students lost one of their family members in the pandemic after getting COVID-19. The mean scores for the coping and personal resilience measures were 18.74 (SD: 5.60) and 3.02 (SD: 0.42), respectively (Table 1).

**Table 1:** Students’ characteristics (*n* = 256).

**Characteristics**	**Categories**	**N**	**Percentage**
Year of study	II Year	88	34.3
	III Year	86	33.5
	IV Year	83	32.3
Exposure as a bedside nurse	Yes	101	39.3
	No	156	60.7
Tested COVID-19 positive	Yes	26	10.1
	No	231	89.9
Students hospitalized^#^	Yes	29	11.3
	No	229	88.7
A family member died^#^	Yes	15	5.8
	No	242	94.2
Quarantine status	Yes	202	78.6
	No	55	21.4
		Mean	SD
Age (years)		21.27	1.19
Personal resilience		3.02	0.42
Coping skills		18.73	5.60
Lockdown/pandemic fatigue scale		31.16	7.05

# *Hospitalization and death due to COVID-19*.

Quarantine (days) Mean (SD, range) 13.27 (2.93, 5–20).

Table 2 shows the students’ responses on the lockdown/pandemic fatigue scale. The mean fatigue scale score was 31.16 (SD: 7.05) out of the maximum possible score of 50. An independent sample t-test shows a significantly higher mean score (*p* = 0.026) on the fatigue scale for students deployed as bedside nurses during the COVID-19 pandemic than another group. Likewise, students who tested positive during the pandemic reported significantly higher mean scores (*p* = 0.003) than their counterparts. Further, it has been reported that second-year class students especially said higher fatigue scores (*p* = 0.001) than third and final-year students. This can be interpreted that higher class students might be experienced enough to handle patients in clinical after their earlier class experiences and hence reported lower fatigue scores compared to second-year students who are a novice and directly deployed to the clinical areas in a serious pandemic situation. Pearson’s correlation coefficient showed a significant negative relationship between age (*r* = −0.125, *p* = 0.046) and personal resilience status (*r* = −0.286, p = <0.01), and lockdown fatigue. However, coping styles showed a positive relationship with lockdown fatigue (*r* = 0.161, *p* = 0.010; Table 2).

**Table 2:** Relationship of lockdown fatigue with characteristics of the students (*n* = 256).

**Characteristics**	**Categories**	**Mean SD**	**Value of *p***
Year of study^a^	II Year	33.48 ± 6.65	0.001^*^
	III Year	30.16 ± 6.84	
	IV Year	29.65 ± 7.08	
Bedside exposure as nurse^b^	Yes	30.69 ± 7.44	0.394
	No	31.46 ± 6.79	
Tested COVID-19 positive^b^	Yes	35.04 ± 7.35	0.003^*^
	No	30.72 ± 6.89	
Hospitalization after COVID-19^b^	Yes	37.00 ± 6.08	0.026^*^
	No	30.99 ± 7.01	
Family member hospitalized after COVID-19^b^	Yes	32.69 ± 6.24	0.215
	No	30.69 ± 7.41	
Family Member died after COVID-19^b^	Yes	34.40 ± 6.60	0.066
	No	30.96 ± 7.04	
Quarantine status^b^	Yes	31.51 ± 6.81	0.127
	No	29.87 ± 7.81	
		Test statistics	Value of p
Age (years)^#^		−0.125^*^	0.046^*^
Personal resilience^#^		−0.286	<0.001^*^
Coping styles^#^		0.161	0.010^**^

a *Analysis of variance.*

b *Independent t-test.*

# *Person r correlation.*

* *p < 0.05;*

** *p < 0.01*.

Findings revealed that a few of the items, *‘*I worry a lot about my personal and family’s safety during this pandemic’ (*m* = 4.25), ‘I have felt sad and depressed as a result of the pandemic’ (*m* = 3.67), and ‘I frequently felt weak or tired as a result of the pandemic’ (*m* = 3.24) reported more frequently by the participants. On the contrary, a few items that received lower attention were ‘I have difficulty falling or staying asleep over thinking about this pandemic’ (*m* = 2.58), ‘I have been losing my interest in doing the usual things I love’ (*m* = 2.60), and ‘I have been experiencing headaches and body pains’ (*m* = 2.61). Further, looking at the agreement status to fatigue items showed that 91.8% of participants responded that they are worried about personal and family members in the pandemic. Likewise, around two-thirds of the participants (74%) reported feeling depressed and sad during the pandemic, and more than half of the participants (52.5%) felt irritated by a pandemic or its related consequences (Table 3).

**Table 3:** Response to lockdown/pandemic fatigue scale (*n* = 256).

**SN**	**Lockdown/Pandemic fatigue scale items**	**Agree**^**#**^	**Disagree**^**#**^	**Mean**	**SD**	**Rank**
1	I worry a lot about my personal and family’s safety during this pandemic.	236 (91.8)	10 (3.9)	4.25	0.80	1
2	I have felt sad and depressed as a result of the pandemic.	190 (74.0)	40 (15.6)	3.67	0.95	2
3	I frequently felt weak or tired as a result of the pandemic.	128 (49.8)	72 (28.0)	3.24	1.05	3
4	I have difficulty concentrating and am distracted easily.	124 (48.2)	92 (35.8)	3.16	1.14	5
5	I have been feeling irritable.	135 (52.5)	80 (31.1)	3.23	1.05	4
6	have difficulty falling or staying asleep over thinking about this pandemic.	71 (27.6)	147 (57.2)	2.58	1.10	10
7	I have been losing my interest to do the usual things I love.	73 (28.4)	150 (58.4)	2.60	1.15	9
8	I have been experiencing a general sense of emptiness	95 (37.0)	114 (44.4)	2.88	1.17	7
9	I have been experiencing headaches and body pains	80 (31.1)	153 (59.5)	2.61	1.20	8
10	I have thoughts that this pandemic will never end soon	106 (41.2)	105 (40.8)	2.95	1.10	6

# *Agree (Agree + strongly agree) & Disagree (Disagree + strongly disagree)*.

Variables that showed a significant association with lockdown fatigue were entered into the multiple linear regression model. The model explained 16.8% in the variance of the lockdown fatigue, which was statistically significant (*F* = 7.170, *p* = <0.001), which indicated the model fit the variables. Out of inserted variables in the model, personal resilience and the second year of the study predicted lockdown fatigue among participants. This can be interpreted as those students who are studying in the lower class (*β* = 3.198, *p* = 0.015) and had poor resilience status (*β* = −4.311, *p* = <0.001) reported higher fatigue during the ongoing pandemic (Table 4).

**Table 4:** Multiple linear regression on factors associated with pandemic fatigue (*n* = 256).

**Variables**	**ß**	**Beta**	**95% CI**	**t-value**	**Value of *p***
Constant	37.604		17.809–57.399	3.742	0.000
Age	0.137	0.023	−0.735–1.009	0.308	0.758
Coping	0.119	0.094	−0.030–0.0.268	1.569	0.118
Resilience	−4.311	−0.259	−6.216−(−2.406)	−4.457	<0.001^*^
Second year	3.198	0.215	0.619–5.779	2.443	0.015^*^
Third year	0.057	0.004	−2.039–2.152	0.054	0.958
Tested COVID-19 positive	2.613	0.112	−0.0477–5.703	1.666	0.097
Hospitalization after COVID-19	3.154	0.073	−2.549–8.857	1.089	0.277

*ß, Standard regression coefficient; CI, confidence interval; F, 7.170, p < 0.001, R square, 16.8%*.

* *Value of p < 0.05*.

## Discussion

The current digital survey was conducted to study the pandemic fatigue and the influence of nursing undergraduates’ socio-demographic variables, coping styles, and resilience in mediating fatigue during the third wave of the COVID-19 pandemic between February and May 2021. The government has taken unprecedented measures to curb the further infection of different variants of the COVID-19 virus from time to time. Further, the authors presumed that the sudden shifting of traditional classroom teaching and clinical learning to virtual learning coupled with home or hostel confinement and reduced physical activities during the COVID-19 pandemic might lead to more fatigue in nursing students (Liu et al., 2021). In the certain phase of the pandemic, nursing students are confined in the hostel or wherever they are, which further intensifies many psychological problems, including anxiety, depression, and fatigue (Brooks et al., 2020). Heavy clinical load, daily infection precautionary measures at work and hostel, reduced social connections, and social distancing could lead to mental fatigue, anxiety, frustration, and boredom (Brooks et al., 2020; Chao et al., 2020; Morales-Rodríguez, 2021).

Participants’ mean fatigue score was 31.16 (SD: 7.05), indicating moderate fatigue status during the ongoing pandemic. A crunch of studies used the lockdown/pandemic fatigue scale; hence, authors find it challenging to compare these findings. However, our study results are in line with a study conducted (Labrague and Ballad, 2021) at Muscat, Oman, on college students who used the pandemic fatigue scale (PFS) reported a similar level of fatigue (31.54; SD: 6.93) during the pandemic. Further, a Chinese study (Liu et al., 2021) reported that more than 67.3% of nursing students said fatigue which is almost doubled (36%) to work conducted (Geiger-Brown et al., 2012) on nurses working in different shifts using the Occupational Fatigue Exhaustion Recovery Scale. Our findings are in consensus with the work (Teng et al., 2020), who reported fatigue in 73.7% of frontline healthcare workers during an initial COVID-19 outbreak in China as measured by the Fatigue Self-Assessment Scale (Teng et al., 2020). These results further follow another work from Australia; a few months after lockdown, the Chalder Fatigue Questionnaire reported a significant level of fatigue (Nitschke et al., 2021). Shreds of evidence from the United States, Saudi Arabia, and India report a substantial amount of tiredness, and increased fatigue, boredom, worry, and loneliness, suggesting some efforts to support the affected population to combat the adverse effect of prolonged lockdown, confinement, and other confinement measures imposed by government time to time (Majumdar et al., 2020; Meo et al., 2020). In contrast, medical students’ fatigue status was relatively low (13.8%) before the COVID-19 outbreak (Abdali et al., 2019). Owing to different measurement tools on fatigue between different studies across the globe indicates cautious interpretations and extrapolating of the findings. Considering the negative impact of fatigue on the individual’s physical, mental, cognitive, and behavioral functions (Trendall, 2000), it is imperative to devise support or other stress-relieving strategies to benefit mental health.

The symptoms of fatigue among nursing undergraduates, lack of concertation, irritation, disturbed sleep, feeling depressed and emptiness, and tired are other symptoms reported by the students. These symptoms are in line with the earlier Indian work conducted on professionals and students who reported various indicators of symptoms, including anxiety, higher stress, feeling of sleeplessness, depression, a safety concern for family, tiredness, and worry for personal safety and other physical discomforts, during the lockdown (Majumdar et al., 2020). The reported fatigue symptomatology in this study was similar to the report of the symptoms presented by Australian Psychological Society (2020), which reported a loss of interest in previously enjoyed activities, physical exhaustion, fear, anxiety, emotional outburst, reduced motivation, depression, and difficulty in focusing and problem-solving as a typical array of symptoms of lockdown fatigue.

Students’ year of study, COVID-19 positive status, and post-COVID-19 hospitalization directly impacted the development of lockdown fatigue. In particular, final-year and third-year students reported lower lockdown fatigue than second-year students. These findings are anticipated, as, during the study, students will be exposed to different clinical conditions and environments to develop positive temperament and adaptive behavior to work with different kinds of patients, which help a student to further deal with such kind of disastrous conditions (Benner, 2004; Sonika and Kumar, 2019). The present study’s findings of lower stress among the higher level of education are also in consensus with the previous studies conducted on nursing students (Kumar and Nancy, 2011; Dharra and Kumar, 2021). Furthermore, previous work on stress among nursing students showed a declining trend as they progress to higher studies, similar to the present report (Kumar and Nancy, 2011; Fornés-Vives et al., 2016). These findings of higher stress in lower-class students indicate developing an evidence-based support system through evidence-based intervention to assist budding nurses in developing coping strategies and adaptative resilience to deal with such disastrous situations effectively.

Further, regression analysis findings showed a significant negative association of personal resilience with lockdown fatigue, suggesting a protective role of individual resilience against the negative consequences of restrictions faced during mandatory home isolation or quarantine during the pandemic. These study findings align with the earlier work that reported a significant negative relationship between personal resilience and lockdown fatigue in college students (Labrague and Ballad, 2021). Close similar findings presented in earlier work reported a positive impact of personal resilience on improving mental health and psychological outcomes among different populations around the globe (Tsay et al., 2001; Charuvastra and Cloitre, 2008; Ran et al., 2020). The study findings draw the mentor and teachers’ attention to building resilience as a strategy to help a student bounce back from such adversity and traumatic situations. To our current knowledge, this is the first of its study in the area, highlighting the impact of individual resilience and coping styles on combating negative consequences on mental health associated with a pandemic, hence supplementing the existing knowledge in this area of research.

Students who used higher coping styles showed higher lockdown fatigue in the current study. Previous studies on nursing students also have identical findings for a significant correlation between fatigue and nursing students’ coping (Nurhidayati et al., 2021). Likewise, another study reported a significant relationship between coping mechanisms and fatigue, suggesting a positive impact of coping mechanisms on fatigue during adverse situations (Michalec et al., 2013). In contrast, the findings on the relationship between coping and fatigue are in non-accordance with many previously published studies that reported a negative relationship between coping mechanisms and fatigue (Tugade and Fredrickson, 2004; Cao et al., 2020; Tull et al., 2020). These contentious findings on coping and fatigue provide additional knowledge to understand the precise mediating effect of coping mechanisms in acute and chronic adversity, including the current pandemic. Instead, higher coping use in students might help reduce fatigue and other mental health issues, including irritation, sleeplessness, distraction, sadness, and body aches compared to students not or less use of coping strategies as mentioned in earlier research (Labrague and De los Santos, 2020; Labrague, 2021; Roberts et al., 2021). However, authors speculate the use of a different tool and longer duration of a pandemic for such unforeseen results on the relationship between coping and fatigue. However, it is vital to devise strategies to improve resilience and coping mechanisms among nursing students to maintain and improve their mental health and overall psychological well-being.

### Limitations

The study should be appraised under many limitations, and other researchers should be cautious while interpreting and extrapolating the results. First, the cross-sectional nature of the survey impedes computing the exact relationship between different variables, including fatigue, coping, and resilience. It is difficult to interpret from the study that higher resilient and coping mechanisms students have lesser fatigue during the lockdown in comparison to counterparts. Hence, the authors recommend using different study designs, including randomized controlled trials or case–control studies, to understand the mediating effect of resilience in improving fatigue and coping skills in students. Second, a study with a higher sample size is recommended to enhance generalizability over other similar populations. Third, online surveys carry inherent bias, including social-desirability bias, and need caution to interpret the findings.

## Conclusion

As per the findings, lockdown-related fatigue was common in nursing undergraduates during the third wave of the COVID-19. Further, the junior students reported tested positive and were hospitalized during the pandemic reported higher fatigue than their counterparts. The study reported that senior students with higher resilience levels said lower fatigue, indicating the significance of perceived professional self-confidence and the need for preparation and precautions to take in a disease outbreak. These findings also reflect the necessity of developing junior students’ professional competencies and patient handling. However, a structured curriculum and adequate clinical exposure will train the students in developing professional competencies and patient handling skills.

### Implications for Nursing Management

Nursing students face enormous challenges during the ongoing pandemic, including the transition of teaching-learning pedagogy, deployment in different levels of clinical assignment, and many other personal and professional obligations. Individual resilience helps beat the negative consequences of pandemic fatigue in nursing students. Nursing educators should prioritize proactive measures to reduce pandemic-induced fatigue and strengthen personal resilience among students. Resilience promotional strategies could be an alternative intervention to improve more use of positive coping strategies to enhance the mental health promotion of nursing students.

## Data Availability Statement

The raw data supporting the conclusions of this article will be made available by the authors, without undue reservation on a individual request of a researcher to the corresponding author.

## Ethics Statement

The studies involving human participants were reviewed and approved by Institutional Ethics Committee, All India Institute of Medical Sciences, AIIMS Rishikesh. The patients/participants provided their written informed consent to participate in this study.

## Author Contributions

RK considered and designed the study, conducted research, provided research materials, and collected and organized data. KB wrote the initial and final draft of the manuscript and provided logistic support. YB analyzed and interpreted data and did the final editing. All authors contributed to the article and approved the submitted version.

## Conflict of Interest

The authors declare that the research was conducted in the absence of any commercial or financial relationships that could be construed as a potential conflict of interest.

## Publisher’s Note

All claims expressed in this article are solely those of the authors and do not necessarily represent those of their affiliated organizations, or those of the publisher, the editors and the reviewers. Any product that may be evaluated in this article, or claim that may be made by its manufacturer, is not guaranteed or endorsed by the publisher.
